# How females think about themselves and how they assume that significant others think about them: The influence of perspective taking on self-referential processing

**DOI:** 10.1371/journal.pone.0217870

**Published:** 2019-05-31

**Authors:** Saskia Doreen Forster, Barbara Drueke, Sara Britz, Siegfried Gauggel, Verena Mainz

**Affiliations:** Institute of Medical Psychology and Medical Sociology, University Hospital of the RWTH Aachen University, Aachen, Germany; Middlesex University, UNITED KINGDOM

## Abstract

People maintain a positive self-concept through positive self-appraisals (*Self-Serving Bias Effect*, SSBE) and a diminished memory for self-threatening information (*Mnemic-Neglect Effect*, MNE). Other people also influence a person’s self-concept. This study investigated SSBE and MNE in 60 females by using a trait-judgment paradigm applying two perspectives (self- and third-person appraisals) and a recall task. Additionally, self-esteem was assessed as an associated factor. SSBE and MNE were found in both kinds of appraisal perspectives. Interestingly, participants saw themselves as even more positive in reflected appraisals. SSBE and self-esteem were associated only in self-appraisals, indicating a larger SSBE on self-appraisals with raising self-esteem. In conclusion, both what females think about themselves and how they assume that others think about them preserve their overall positive self-concept.

## Introduction

A stable and positive self-concept is fundamental to psychological functioning and behavior. Metacognitive or self-referential knowledge, i.e., reflections on and knowledge about one's own abilities, characteristics, sensations, attitudes, and preferences is necessary in many everyday situations in order to be able to act in a targeted and socially appropriate manner. For example, people often rely on their self-knowledge when inferring others’ mental states in social interactions [1]. In recent years, various empirical studies have investigated specific processes related to the self, in order to develop models of how people arrive at conclusions about themselves (e.g., [2]). These studies examined different aspects of self-referential processing, including ownership [3], the processing of self-related cues [4], autobiographic memory [5], the accuracy of meta-perception regarding the self [6], or individual judgments of personality traits [7, 8]. According to these studies, self-referential processes differ from other forms of information processing, such as references to other people’s mental states or semantic processing [9].

Research studies have emphasized the importance of different perspectives and the influence of perspective taking on a person’s self-concept. According to the symbolic interactionist perspective, the *self* is created through social interactions [10]. It is undeniable that knowing one’s self, for example one’s character traits, depends to a certain extent on what the person knows about and how he or she experiences other people. Whether people consider themselves to be *attractive* or *unattractive*, *skillful* or *unskillful* depends at least partially on social comparisons [11]. The research investigating the magnitude and accuracy of self-knowledge by assessing the overlap between different people’s views on a person’s self-concept has investigated the impact of social interactions on a person’s self-knowledge [12]. Findings highlight the extent to which an individual’s view of his or her own personality accurately reflects other people’s perspectives (e.g. [12, 13]). Although there is an overlap between one’s own and other people’s appraisals of themselves, the correlation between self-knowledge and other people’s knowledge about a person is not as strong as the symbolic interactionist framework originally assumed [12]. One reason for this discrepancy might be that how important an individual considers the other person to be and how close an individual feels to that person determines whether this individual takes the other person’s opinion into account [14, 15]. In addition, other researchers have concluded that a person’s self-concept is not so much influenced by what others actually think, but instead by what the person assumes that other people think (i.e., reflected appraisals), for example, in situations in which people do not receive direct feedback from other people [14, 15]. In line with this position, our own research focuses on an individual’s self-knowledge by investigating the influence of perspective taking on trait-judgments and memory processes with regard to the self. Two effects have been repeatedly found in different experimental studies on self-referential processing of personality traits, namely the so-called *Self-Serving Bias Effect* (SSBE) and the *Mnemic-Neglect Effect* (MNE).

The SSBE is a well-known phenomenon in social and personality psychology. It can be observed in healthy participants while they are judging certain information as *self-describing* or *non-self-describing* [16–18]. An SSBE occurs when people attribute more positive than negative information to themselves [7, 19]. Studies of SSBE have mostly focused on the number of different kinds of choices that participants make. Reaction times (RTs) and how they are associated with SSBE have rarely been investigated, although they could provide additional information about the mechanisms underlying SSBE. Usually, shorter RTs are required when a person judges self-related stimuli as compared to stimuli with no self-relevance [20]. Therefore, self-referential biases would be expected when RTs are being measured as the dependent variable. Specifically, we would expect that shorter RTs would occur when participants attribute positive stimuli to themselves and when they reject negative stimuli [21, 22]. Conversely, it should take longer to reject positive and accept negative personality relevant information. In this article, we refer to this effect on RTs as the *Self-Serving Reaction Time Effect* (SSRTE).

The MNE refers to an effect that occurs during recall tasks. It reflects a memory advantage for positive compared to negative self-relevant information [23, 24]. Some studies have investigated whether the degree to which MNE occurs can be influenced by the use of certain recall procedures or strategies, detecting that the MNE differs in intensity depending on the length of the recall period and the record procedures used during the recall [25]. Additional strategies that individuals use might also influence their recall performance. For example, it is not yet known to what extent either the self-relevance of stimuli or stimulus valence facilitates recall. Previous experimental evidence suggests, however, that the SSBE and MNE effects are quite robust, and that they occur regardless of participants’ age, gender, psychopathology, or culture [26, 27].

Until now, there have been only a few experimental studies that have examined biases in judgments of one's own character traits while taking a third-person's perspective [28–31]. Importantly, the main focus of these studies was on the underlying neuronal correlates of direct versus indirect self-referential information processing, in which first- and third-person appraisals were compared. These imaging studies indicated that both self-appraisals and reflected appraisals involve distinct regions in the medial prefrontal cortex [28]. Furthermore, the perceived closeness between a person and the other person whose perspective was being taken appeared to modulate the neural basis of SSBE in the reflected appraisals [29]. However, behavioral results, including the specific influence of the third-person perspective on SSBE and MNE were not reported in detail, and no conclusions can be drawn about the psychological impact of different appraisal perspectives.

The perspective that one takes seems to be an important mechanism involved in self-judgments. The thereby affected self-concept and self-knowledge, i.e., an “individual’s beliefs about himself or herself” ([32] p247) are closely intertwined with one’s self-worth and self-esteem, which are understood as an individual’s “broadest self-evaluation” ([32] p248). People strive to develop and maintain their self-esteem and a positive self-concept in order to fulfill their own needs and motivations [33]. The ascription of positive traits to one’s self is self-reinforcing and it results in *self-enhancement*. At the same time, the rejection of negative traits as applicable to one’s self is a form of *self-protection* [33, 34]. Hence, whereas the SSBE is understood to be both self-protective and self-enhancing, the MNE is understood only as a self-protective process [23, 24]. Both self-enhancement and self-protection help a person to maintain positive self-esteem and a positive self-concept [35].

Asencio [36] found that a person’s self-esteem seems to moderate the influence that other people’s assumed opinions can have on that person’s own self-concept. This means that people with higher self-esteem are more likely to share a significant other person’s assumed perspective about themselves than are people with lower self-esteem. Also, people with higher self-esteem are likely to interpret another person’s reflected appraisal of themselves as consistent with their self-concept, even if their own and the other person’s views are incompatible (e.g., someone thinks of himself as a nice person and assumes that other people also think of him as nice, although the other person actually does not [36]). Consistent with Rosenberg [14] and Hoyle et al. [15], Asencio [36] found that the impact of reflected appraisals of highly valued and trusted others on one’s self-view is greater than that of less valued and less trusted people.

The present study investigated differences among SSBE, SSRTE, and MNE by comparing trait-judgments taken from one's own perspective (self-appraisals) with trait-judgments taken from the perspective of a significant other (reflected appraisals). The reflected perspective refers to a significant other, because research has shown that significant others are most influential with regard to one's own self-concept [14, 15, 36]. Importantly, the present study did not investigate the accuracy of the self-appraisals. Because we did not include an external criterion for the validity of the judgments, they remained subjective. Moreover, because previous studies have shown that men and women differ in both self-reported perspective taking [37] and self-esteem [38], we included only healthy females in this study. Doing so allowed us to minimize random error in identifying the differences between self-appraisals and reflected appraisals. Regarding the bias effects, our hypotheses were as follows: We hypothesized that people would assign more positive adjectives and fewer negative adjectives as self-describing (SSBE). Moreover, we predicted that RTs would be shorter while assigning positive adjectives as self-describing and negative adjectives as non-self-describing compared to rejecting positive adjectives as self-describing and assigning negative adjectives as self-describing (SSRTE). Finally, we expected that recall for positive, self-describing adjectives would be better than recall for negative self-describing adjectives (MNE). Although a certain amount of overlap in choices between self-appraisals and reflected appraisals was expected, the present study was especially interested on the differences that would emerge when the perspective changed, i.e., the effect of two different perspectives on SSBE, SSRTE, and MNE. The focus of the research, therefore, was on the differences between how females think about themselves and how they assume that significant others think about them. Finally, we investigated the associations among self-esteem, self-appraisals, and reflected appraisals. Consistent with the view of Asencio [36] that people with high self-esteem rather share their reflected appraisals of other people who are close to them, we expected to find negative associations among participants’ self-esteem and the different appraisal perspectives, i.e., higher self-esteem was expected to be associated with a smaller discrepancy between self-appraisals and reflected appraisals.

## Methods

### Participants and procedure

The sample size was estimated based on an a priori power analysis using the statistical software G*Power (Version 3.1, [39]). For the planned 2 x 2 within-subject repeated-measures design (see section on data analyses), the calculated a priori power analysis following the instructions of Rasch, Friese, Hofmann and Naumann [40] produced a required sample size of 24 participants for a power of .80, an alpha-error of .05, a moderate effect size, and moderate correlations among the measures. For the planned regression analysis (see section on data analyses), the calculated a priori power analysis following the instructions of Faul, Erdfelder, Buchner and Lang [41] produced a required sample size of 55 participants, with a power of .80, an alpha-error of .05, and a moderate effect size.

Sixty female participants (age: *M* = 23.7; *SD* = 4.4, range 18–47) who were native speakers of German with no history of mental or neurological disease were recruited via flyers and word of mouth. The inclusion criteria were stated both in the flyer and via e-mail contact with each participant. All participants received verbal and written explanations of the purpose and procedures of the study, and prior to participation, they gave written informed consent in accordance with the Declaration of Helsinki. However, the specific hypotheses of the study were not communicated to the participants. Subsequently, each participant was randomly assigned to one of the testing-conditions (see section on trait-judgment task for a detailed description of the testing-conditions). All participants were tested individually in a single session that lasted approximately 45 minutes. Each participant was guided through the course of the study by an experimenter whom was present during the investigation and instructed each participant on the individual task requirements before each task was performed. Short task instructions were moreover written out on the computer screen. After the session, each participant received a small monetary compensation. The ethics committee of the Medical faculty of the RWTH Aachen University approved the study (EK100/17).

### Self-referential processing tasks

To investigate self-referential information processing and to further explore the SSBE, SSRTE, and MNE, the performance during two consecutive tasks (a trait-judgment task and a trait-recall task) was assessed. The two tasks were performed using Presentation software (Version 19.0 11.14.16, Neurobehavioral Systems, Inc., Berkeley, CA, www.neurobs.com).

#### Materials

For the trait-judgment task, one set of 20 and another set of 50 trait adjectives (half of which were classified as *positive* and half as *negative*) were selected from the Aachen List of Trait Words [42]. Several stimulus properties were controlled for in the second set of 50 trait adjectives (because the first set was not relevant for further analyses; see section on trait-judgment task) in order to rule out systematic biases in participants’ choices and their memory [43, 44]. To test whether the 25 positive and 25 negative adjectives in the second set differed with respect to the word length, word frequency, absolute value of the valence, and absolute value of social desirability, a multivariate factorial analysis of variance (MANOVA) was performed using post hoc univariate analyses. The MANOVA revealed no significant main effect [*F*(4,45) = 0.37, *p* = .83]. All post-hoc univariate comparisons between positive and negative adjectives were nonsignificant (all *p*s > .05), indicating that there was no difference between positive and negative trait adjectives with respect to word length, word frequency, absolute value of the valence, or the absolute value of social desirability. However, the direction of the value of the valence and the value of social desirability obviously differed between the positive and the negative adjectives.

#### Trait-judgment task

In the first task, all 70 trait adjectives were randomly presented in a white color on a black screen for 2500 ms each, and were preceded by a variable fixation cross (presented between 500 und 800 ms). Before the 50 test trials began (consisting of 25 positive and 25 negative trait adjectives) and after they had been presented, 10 trials (each consisting of five positive and five negative trait adjectives) were presented as training and to minimize primacy and recency effects. These trials were excluded from the analyses. Participants were instructed to decide for each adjective that was presented whether it was self-describing or non-self-describing by pressing one of two buttons (Yes–No) on a keyboard using either their index or their middle finger. A forced-choice paradigm and dichotomous coding were required in order to assess the effect of memory on the subsequent recall task. The order in which the fingers were used to answer the question (Yes–No) was fixed for right-handed and for left-handed participants. Each participant was randomly assigned to one of the testing-conditions, and each started with either the SELF or the OTHER testing-condition. Each participant performed the trait-judgment task twice: Once using her own perspective (SELF) and once while assuming the perspective of a significant other (OTHER), i.e., assuming what that person’s appraisal would be (reflected appraisal). The significant other was identified through short instructions immediately before the OTHER-condition, and participants were reminded orally to retain the perspective of this specific significant other. Brief instructions concerning the judgments made from the first-person perspective were provided just before the SELF-condition began. To facilitate maintenance of the OTHER-perspective, the name of the predefined significant other was written above the trait adjective that was presented and which was to be judged (e.g., “Tina thinks I am …”). Under the SELF-perspective, participants were reminded of the first person-perspective with the words “I am …”, which were written directly above each trait adjective that was to be judged.

The percentage of positive and negative adjectives that were chosen as self-describing or non-self-describing served as the dependent variables in the task that assessed the SSBE. Percentages were chosen instead of absolute values in order to depict the proportion of adjectives that had been chosen, as participants differed in the number of judgments they had missed. Additionally, choice dependent RT differences in the context of the SSBE were further examined to assess SSRTE. In the analyses, only those participants who were less than two standard deviations (SD) from the mean on missing values were included; this resulted in a total of 57 participants.

#### Trait-recall task

After completing the trait-judgment task, participants were asked to recall as many of the adjectives that had been presented as possible and to repeat them orally in any order. For completing this task, participants were allowed a fixed interval of five minutes. The oral answers were voice recorded (using Presentation software, Version 19.0 11.14.16, Neurobehavioral Systems, Inc., Berkeley, CA, www.neurobs.com) and simultaneously written down by the experimenter.

The dependent variables to assess MNE were the percentage of positive and negative self-describing and non-self-describing adjectives recalled. Percentages were used instead of absolute numbers because participants differed in the number of missed judgments during the trait-judgment task.

### Self-esteem

To assess participants’ self-esteem, an adapted German version of the Rosenberg Self-Esteem Scale (RSES) from von Collani and Herzberg [45] was used. Using this scale, participants were presented with 10 items, which they were asked to answer on a 5-point Likert scale that ranged from *strongly disagree* (1) to *strongly agree* (5). The maximum score was 50, which indicated very high self-esteem. The participants completed the RSES prior to completing the trait-judgment and recall tasks. To assess reliability, Cronbach’s alpha was calculated as a measure of internal consistency. With a Cronbach’s alpha of .87, the internal consistency of this questionnaire was satisfactory. For the following analyses, sum scores were calculated.

### Data analyses

All data were analyzed using R x64 3.4.2 [46]. Participants with missing values greater than two standard deviations from the group mean were considered outliers, and they were excluded from all of the analyses. In the analyses of RTs and the recall task, only those participants were included who had made at least one choice in all of the conditions in the judgment task. Participants with missing values resulting from not having made a choice were excluded from these analyses. The *N*s that resulted after these exclusions are shown in the tables with the analyses. For all of the analyses of variance (ANOVAs), partial eta-squared (*η*^*2*^_*P*_*)* is shown as effect sizes with 90% confidence intervals (CI). Additionally, generalized eta-squared (*η*^*2*^_*G*_) is reported as the effect size [47], because Bakeman [48] recommended this for repeated-measures analyses. All post hoc analyses are reported with Bonferroni corrections. Only Bonferroni-corrected *p*-values are reported for the post hoc analyses. All of the data for the present analyses are available online (https://osf.io/t8cx6/; doi: 10.17605/OSF.IO/T8CX6).

#### Order effects

To control for potential task order effects, two MANOVAs were performed to compare RTs and recall scores for participants who started with the SELF testing-condition (followed by the OTHER testing-condition) with the participants who started with the OTHER testing-condition (followed by the SELF testing-condition). Both MANOVAs revealed no significant main effect [RT: *F*(8,48) = 0.92, *p* = .51; recall-performance: *F*(8,48) = 0.55, *p* = .81]. All post hoc univariate comparisons between the participants who started with the different testing-conditions were nonsignificant (*p*s >.05).

#### Trait-judgment task: SSBE and SSRTE

To assess the SSBE, 2 x 2 repeated-measures analyses of variance (ANOVAs) were performed in which the valence of the adjectives (positive, negative) and the testing-condition (SELF, OTHER) were the within-subject factors, and the percentages of self-describing positive and negative adjectives and the percentages of non-self-describing positive and negative adjectives were, respectively, the dependent variables. All of the post hoc pairwise comparisons are reported in the results section.

To assess the SSRTE, 2 x 2 repeated-measures ANOVAs were conducted in which the valence of the adjectives (positive, negative) and the testing-condition (SELF, OTHER) were the within-subject factors, and RTs to the self-describing and non-self-describing positive and negative adjectives were, respectively, the dependent variables. All post hoc pairwise comparisons are reported in the results section on the SSRTE variables.

#### Trait-recall task: MNE

To evaluate the MNE, a 2 x 2 x 2 repeated-measures ANOVA was conducted in which the valence of the adjectives (positive, negative) and the allocation of the adjectives (self-describing and non-self-describing adjectives) and the testing-condition (SELF, OTHER) were the within-subject factors and the percentages of self-describing and non-self-describing positive and negative adjectives were the dependent variables. Post hoc pairwise comparisons are shown in the results section for the MNE variables.

#### Recall advantages and strategies

Two additional analyses were conducted to analyze potential recall advantages and strategies. First, using a descriptive approach, the frequencies of self-describing and non-self-describing positive and negative adjectives that each participant recalled multiple times were recorded and the total number was calculated across all participants (Σ_r>1_: number of words recalled more than once across all participants). The assumption was that the more frequently an adjective was recalled, the greater the accessibility would be.

In a second analysis, associations among recall position, self-appraisals, and valence were evaluated. This analysis was exploratory; however, a potential strategy could be that, for example, a person first recalls positive self-describing traits (thereby self-enhancing and not self-threatening) and only after negative non-self-describing traits (i.e., not self-threatening). We evaluated this possibility as follows. First, for each participant, the self-describing and non-self-describing appraisals and the valence of each adjective were assigned a recall position (range: 1st-40th). For each participant who recalled fewer than 40 adjectives, the recall positions were expanded to 40 in order to have ranks of equal length across the participants. For the adjective that each participant recalled last (for those who recalled fewer than 40 adjectives), the rank that was assigned was 40. The other ranks (1–39) were filled in with the adjectives nearest to the recall position (nearest neighbor interpolation, [49]). In the next step, the percentages of self-describing and non-self-describing positive and negative adjectives for each recall position was calculated across all participants. To determine whether the assigned percentages were associated with the rank orders of each individual’s memory span, Spearman’s rank correlations were calculated to determine the associations between the memory ranks and the percentage of positive self-describing, negative self-describing, positive non-self-describing and negative non-self-describing adjectives, respectively. Following the strategy just described, a negative correlation between the mean percentage of positive self-describing adjectives and the memory ranks (i.e., a low memory rank for a high percentage of positive self-describing adjectives) indicated better access to non-threatening and positively valenced information at the beginning of the recall task.

#### Differences between SELF-perspective and OTHER-perspective: Trait-judgment task

In order to gain better insight into the impact of the perspective on the appraisals, we further examined differences with regard to the perspective from which the judgment had been made. First, the difference between the number of times that positive versus negative adjectives had been chosen as self-describing versus non-self-describing (Diff_positive_: positive self-describing–positive non-self-describing; Diff_negative_: negative self-describing–negative non-self-describing) was calculated for each participant in each testing-condition (SELF, OTHER). Differences greater zero indicated that more adjectives had been chosen as self-describing whereas differences smaller than zero indicated that more adjectives had been chosen as non-self-describing. Second, univariate ANOVAs with the testing-condition (SELF, OTHER) as the within-subject factor and Diff_positive_ and Diff_negative_ as the dependent variables were conducted in order to identify the relative differences between the choices made within the two testing-conditions.

#### Differences between SELF-perspective and OTHER-perspective: Trait-recall task

Next, we examined whether adjectives that had been assigned differently depending on whether they were perceived as self-describing or non-self-describing in the SELF and OTHER testing-conditions were also related to differences in the recall task. For example a participant might have chosen a certain adjective (e.g., *nice*) as self-describing in the SELF-condition but not in the OTHER-condition. To evaluate this possibility, the percentages of recalled adjectives that were assigned equally or differently in the two testing-conditions (SELF, OTHER) were calculated. Second, a 2 x 2 repeated-measures ANOVA was performed with these percentages as the dependent variables and the valence of the adjectives (positive, negative) and the equality of the judgment in the testing-conditions (equal, different) as within-subject factors.

#### Association between self-esteem and choice

To examine the association between self-esteem and choices made during the trait-judgment task, several regression analyses were run separately for the SELF and OTHER testing-conditions in which the sum scores of the RSES were the predictor variable. The criterion variables were the percentages of self-describing positive and negative adjectives and the percentages of non-self-describing positive and negative adjectives in the SELF and OTHER testing-conditions.

## Results

**Fig 1:**
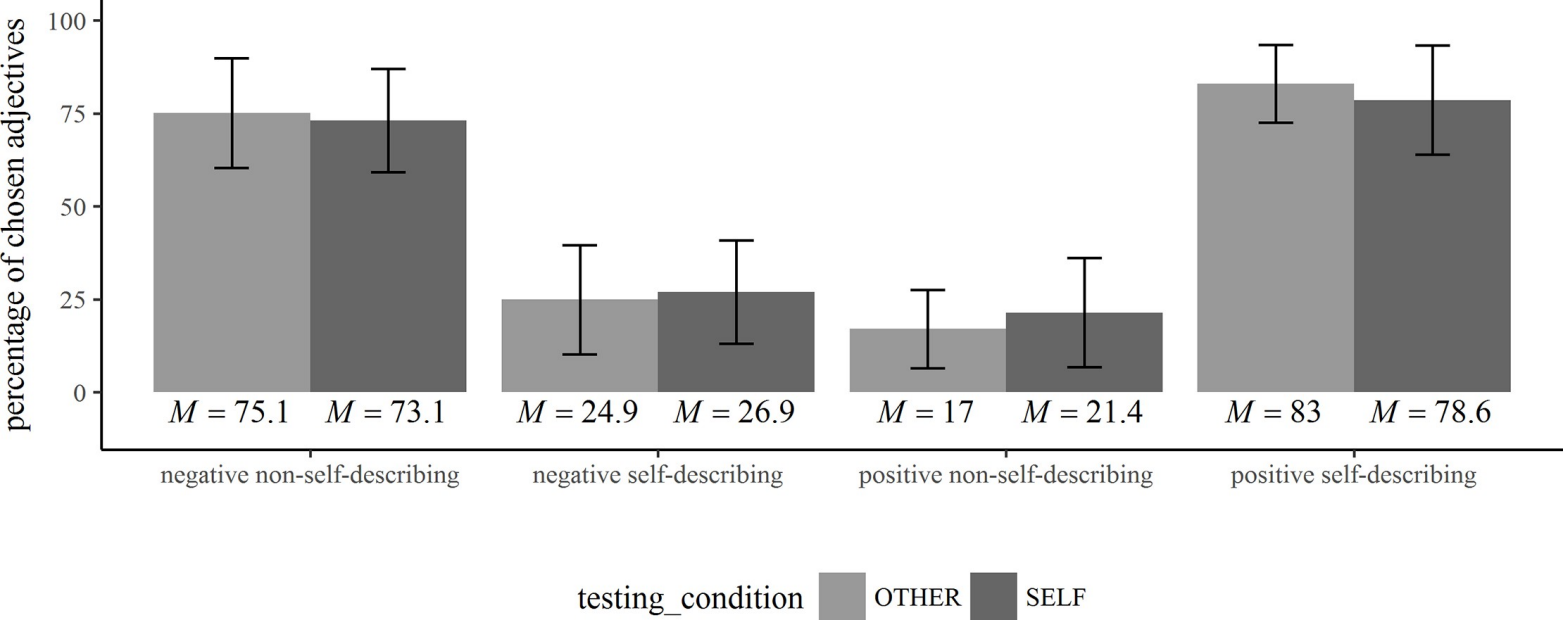
Participants’ choices in the trait-judgment task. Means and standard deviations of the percentage of chosen adjectives in the trait-judgment task (*N* = 57).

**Fig 2:**
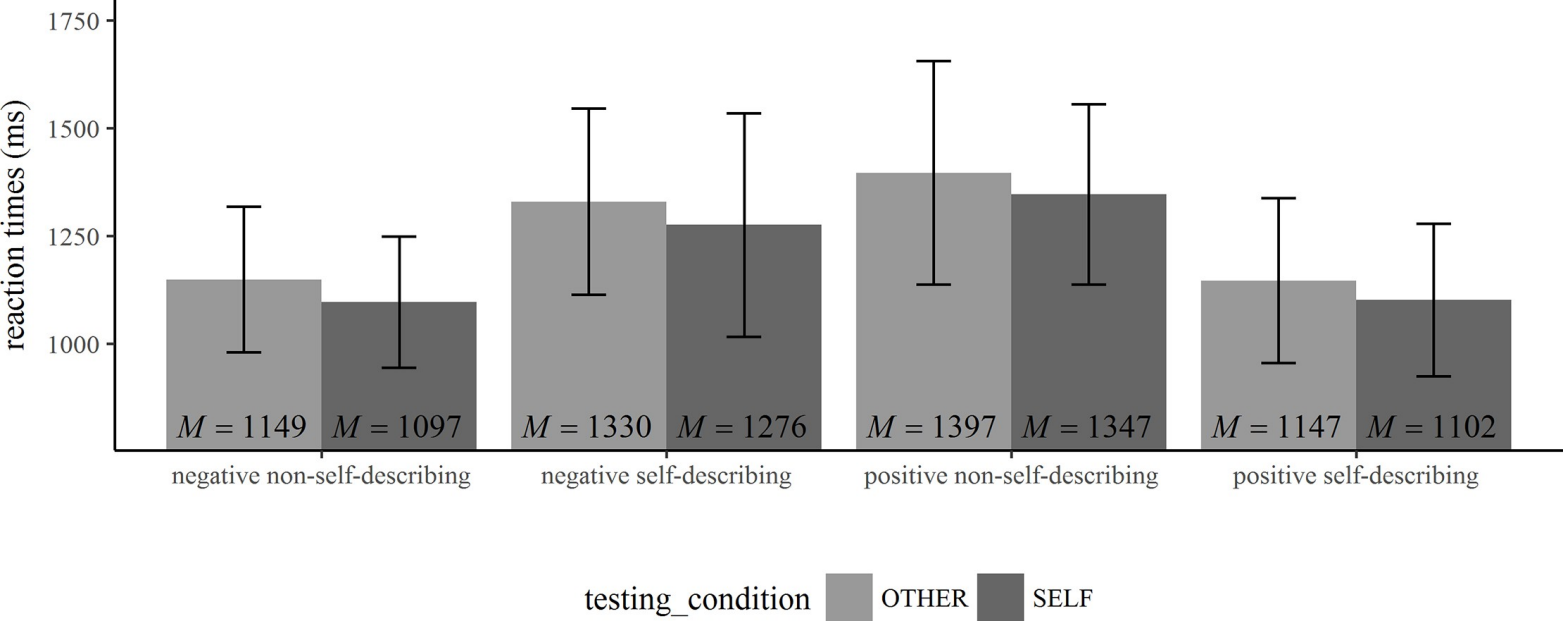
Reaction times in the trait-judgment task. Means and standard deviations of reaction times while classifying the adjectives in the trait-judgment task (*N* = 50).

**Fig 3:**
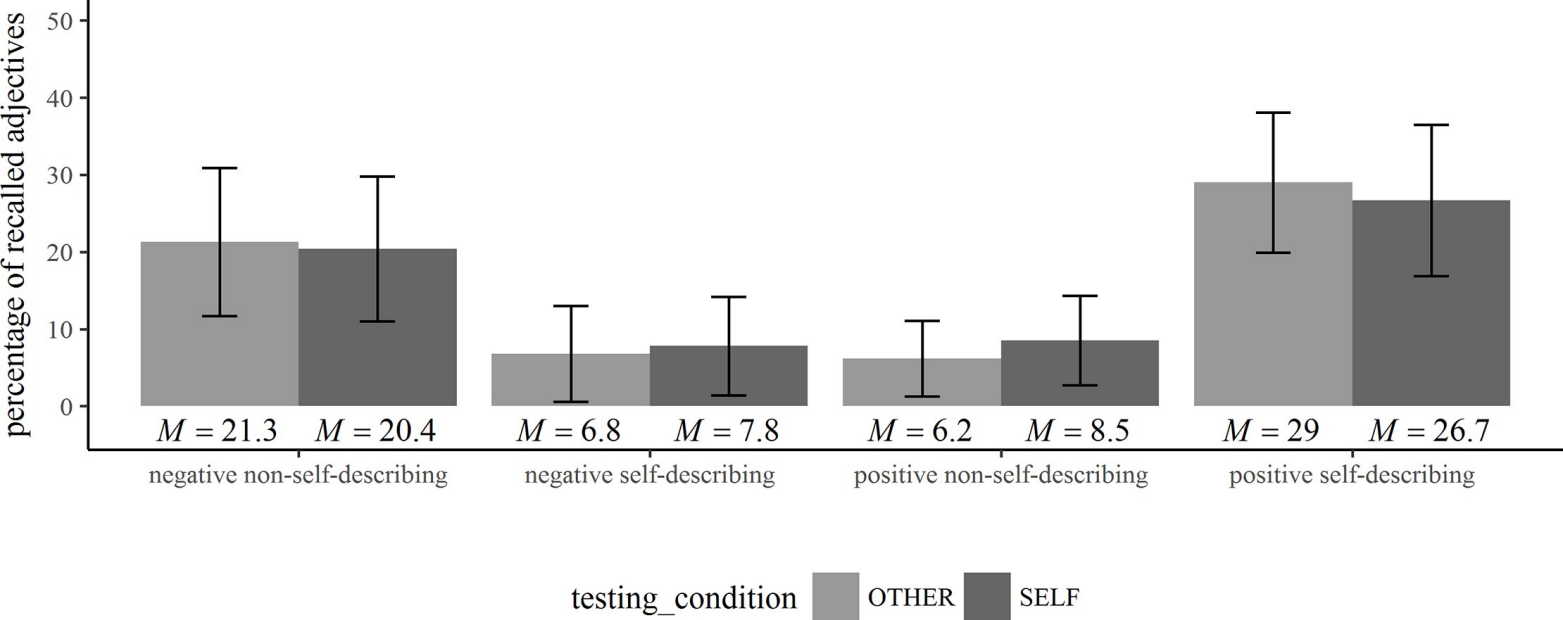
Recall performance in the trait-recall task. Means and standard deviations of the percentage of recalled adjectives in the trait-recall task (*N* = 50).

The means and standard deviations of the participants’ task performance (percentage of chosen adjectives, reaction times, percentage of adjectives recalled) are shown in Tables 1 and 2 and in Figs 1–3.

**Table 1:** Choices and reaction times in the trait-judgment task.

Total numbers and percentages of chosen adjectives (trait-judgment task, *N* = 57)										
							Testing-condition			
							SELF		OTHER	
							Valence			
							Pos^*a*^	Neg^*a*^	Pos^*a*^	Neg^*a*^
Allocation	Testing-Condition	Valence	*M* *(total)*	*SD* *(total)*	*M* *(%)*	*SD* *(%)*	*p*^*1*^	*p*^*1*^	*p*^*1*^	*p*^*1*^
self-describing	SELF	POS^*a*^	19.5	3.7	78.6	14.7		< .001	.12	< .001
		NEG^*a*^	6.7	3.5	26.9	13.9	< .001		< .001	1
	OTHER	POS^*a*^	20.6	2.6	83	10.5				< .001
		NEG^*a*^	6.2	3.7	24.9	14.7			< .001	
non-self-describing	SELF	POS^*a*^	5.3	3.6	21.4	14.7		< .001	.12	< .001
		NEG^*a*^	18.2	3.5	73.1	13.9	< .001		< .001	1
	OTHER	POS^*a*^	4.2	2.6	17	10.5				< .001
		NEG^*a*^	18.7	3.7	75.1	14.7			< .001	
Reaction times in ms (trait-judgment task, *N* = 50)										
							Testing-condition			
							SELF		OTHER	
							Valence			
							Pos^*a*^	Neg^*a*^	Pos^*a*^	Neg^*a*^
Allocation	Testing-Condition	Valence	*M* *(ms)*	*SD* *(ms)*			*p*^*1*^	*p*^*1*^	*p*^*1*^	*p*^*1*^
self-describing	SELF	POS^*a*^	1102	177				< .001	.17	< .001
		NEG^*a*^	1276	259			< .001		< .001	.99
	OTHER	POS^*a*^	1147	191						< .001
		NEG^*a*^	1330	216					< .001	
non-self-describing	SELF	POS^*a*^	1347	209				< .001	1	< .001
		NEG^*a*^	1097	152			< .001		< .001	.04
	OTHER	POS^*a*^	1397	259						< .001
		NEG^*a*^	1149	169					< .001	

*Note*. Total number and percentage of chosen adjectives, RTs, and results from post hoc analyses of SSBE and SSRTE in the trait-judgment task.

^*1*^ Bonferroni-corrected *p*-values

^*a*^ POS: positive, NEG: negative.

**Table 2:** Summary of recall performance in the trait-recall task.

Total number and percentage of recalled adjectives (trait-recall task, *N* = 50)														
							Testing-condition							
							SELF				OTHER			
							Allocation							
							self-describing		non-self-describing		self-describing		non-self-describing	
							Valence							
							POS^*a*^	NEG^*a*^	POS^*a*^	NEG^*a*^	POS^*a*^	NEG^*a*^	POS^*a*^	NEG^*a*^
Testing-condition	Allocation	Valence	*M (total)*	*SD (total)*	*M* *(%)*	*SD* *(%)*	*p*^*1*^	*p*^*1*^	*p*^*1*^	*p*^*1*^	*p*^*1*^	*p*^*1*^	*p*^*1*^	*p*^*1*^
SELF	self-describing	POS^*a*^	6.6	2.4	26.7	9.8		< .001	< .001	.01	.53	< .001	< .001	.1
		NEG^*a*^	1.9	1.6	7.8	6.4	< .001		1	< .001	< .001	1	1	< .001
	non-self-describing	POS^*a*^	2.1	1.4	8.5	5.8	< .001	1		< .001	< .001	1	.45	< .001
		NEG^*a*^	5.1	2.3	20.4	9.4	.01	< .001	< .001		< .001	< .001	< .001	1
OTHER	self-describing	POS^*a*^	7.2	2.3	29	9.1						< .001	< .001	.001
		NEG^*a*^	1.7	1.6	6.8	6.2					< .001		1	< .001
	non-self-describing	POS^*a*^	1.5	1.2	6.2	4.9					< .001	1		< .001
		NEG^*a*^	5.3	2.4	21.3	9.6					.001	< .001	< .001	

*Note*. Total number and percentage of recalled adjectives and post hoc analyses for the MNE in the trait-recall task.

^*1*^ Bonferroni-corrected *p*-values

^*a*^ POS: positive, NEG: negative.

### Trait-judgment task: SSBE and SSRTE

#### SSBE

The repeated-measures ANOVAs to evaluate the proportion of positive and negative adjectives chosen as self-describing and the positive and negative adjectives chosen as non-self-describing, respectively, revealed a significant main effect for the valence of the adjectives [F(1,56) = 518.26, *p* < .001, *η*^*2*^_*P*_ = .9, 90% CI(.86,.92), *η*^*2*^_*G*_ = .81] and a significant interaction between valence and testing-condition [F(1,56) = 5, *p* = .03, *η*^*2*^_*P*_ = .08, 90% CI(.004,.21), *η*^*2*^_*G*_ = .01]. The main effect for testing-condition was not significant [F(1,56) = 1.39, *p* = .24, *η*^*2*^_*P*_ = .02, 90% CI(0,.12), *η*^*2*^_*G*_ = .00]. This indicates that more positive adjectives were selected as self-describing whereas more negative adjectives were selected as non-self-describing (see Fig 1). These findings were more pronounced during reflected appraisals than during self-appraisals. Post hoc pairwise comparisons between the group means are shown in Table 1.

#### SSRTE

The repeated-measures ANOVAs for the RTs in the judgment task for self-describing positive and negative adjectives and non-self-describing positive and negative adjectives revealed significant main effects for the valence of the adjectives (self-describing adjectives: *F*(1,49) = 102.96, *p* < .001, *η*^*2*^_*P*_ = .68, 90% CI(.54,.75), *η*^*2*^_*G*_ = .15; and non-self-describing adjectives *F*(1,49) = 108.22, *p* < .001, *η*^*2*^_*P*_ = .69, 90% CI(.56,.76), *η*^*2*^_*G*_ = .28) and for the testing-condition (self-describing adjectives: *F*(1,49) = 4.19, *p* = .046, *η*^*2*^_*P*_ = .08, 90% CI(.001,.21), *η*^*2*^_*G*_ = .01; and non-self-describing adjectives: *F*(1,49) = 4.8, *p* = .03, *η*^*2*^_*P*_ = .09, 90% CI(.004,.23), *η*^*2*^_*G*_ = .02). There was no significant interaction between the valence of the adjectives and the testing-condition (self-describing adjectives: *F*(1,49) = 0.05, *p* = .82, *η*^*2*^_*P*_ = .00, 90% CI(0,.05), *η*^*2*^_*G*_ = .00; non-self-describing adjectives: *F*(1,49) = 0.01, *p* = .94, *η*^*2*^_*P*_ = .00, 90% CI(0,.01), *η*^*2*^_*G*_ = .00). This indicates that the participants selected positive traits faster than negative traits as self-describing and negative traits faster than positive traits as non-self-describing. It further shows that participants needed more time to decide during the reflected appraisals. To further illustrate the pattern of RTs in the two testing-conditions, means and standard deviations of the RTs are displayed in Fig 2. Post hoc pairwise comparisons between the group means are shown in Table 1.

### Trait-recall task: MNE

The repeated-measures ANOVA for the MNE to assess the effects of the self-describing positive and negative adjectives and non-self-describing positive and negative adjectives revealed significant main effects for the valence of the adjectives [*F*(1,49) = 22.71, *p* < .001, *η*^*2*^_*P*_ = .32, 90% CI(.14,.46), *η*^*2*^_*G*_ = .05], the allocation of the adjectives [*F*(1,49) = 11.29, *p* = .002,*η*^*2*^_*P*_ = .19, 90% CI(.05,.34), *η*^*2*^_*G*_ = .05] and a significant interaction between the valence and the allocation of the adjectives [*F*(1,49) = 284.21, *p* < .001, *η*^*2*^_*P*_ = .85, 90% CI(.78,.89), *η*^*2*^_*G*_ = .54)]and a significant interaction between the valence and the allocation of the adjectives and the testing-condition [*F*(1,49) = 7.73, *p* = .008, *η*^*2*^_*P*_ = .14, 90% CI(.02,.28) *η*^*2*^_*G*_ = .01]. The interaction is shown graphically in Fig 3. There was no significant main effect for testing-condition [*F*(1,49) = 0.55, *p* = .46, *η*^*2*^_*P*_ = .01, 90% CI(0,.1), *η*^*2*^_*G*_ = .00] and no significant interaction between the valence of the adjectives and the testing-condition [*F*(1,49) = 0.38, *p* = .54, *η*^*2*^_*P*_ = .01, 90% CI(0,.09), *η*^*2*^_*G*_ = .00] nor between the allocation of the adjectives and the testing-condition [*F*(1,49) = 1.14, *p* = .29, *η*^*2*^_*P*_ = .02, 90% CI(0,.13), *η*^*2*^_*G*_ = .00]. The results indicated that the positive self-describing and negative non-self-describing adjectives were recalled better than the positive non-self-describing and negative self-describing adjectives. Post hoc pairwise comparisons between the group means are shown in Table 2.

#### Recall advantages and strategies

The analysis of the frequencies of self-describing and non-self-describing positive and negative adjectives that were recalled multiple times revealed that most often positive adjectives previously allocated as self-describing were recalled more than once (Σ_r>1_ = 48), followed by negative non-self-describing adjectives (Σ_r>1_ = 20). Less frequently recalled were positive non-self-describing (Σ_r>1_ = 10) and negative self-describing adjectives (Σ_r>1_ = 5).

**Fig 4:**
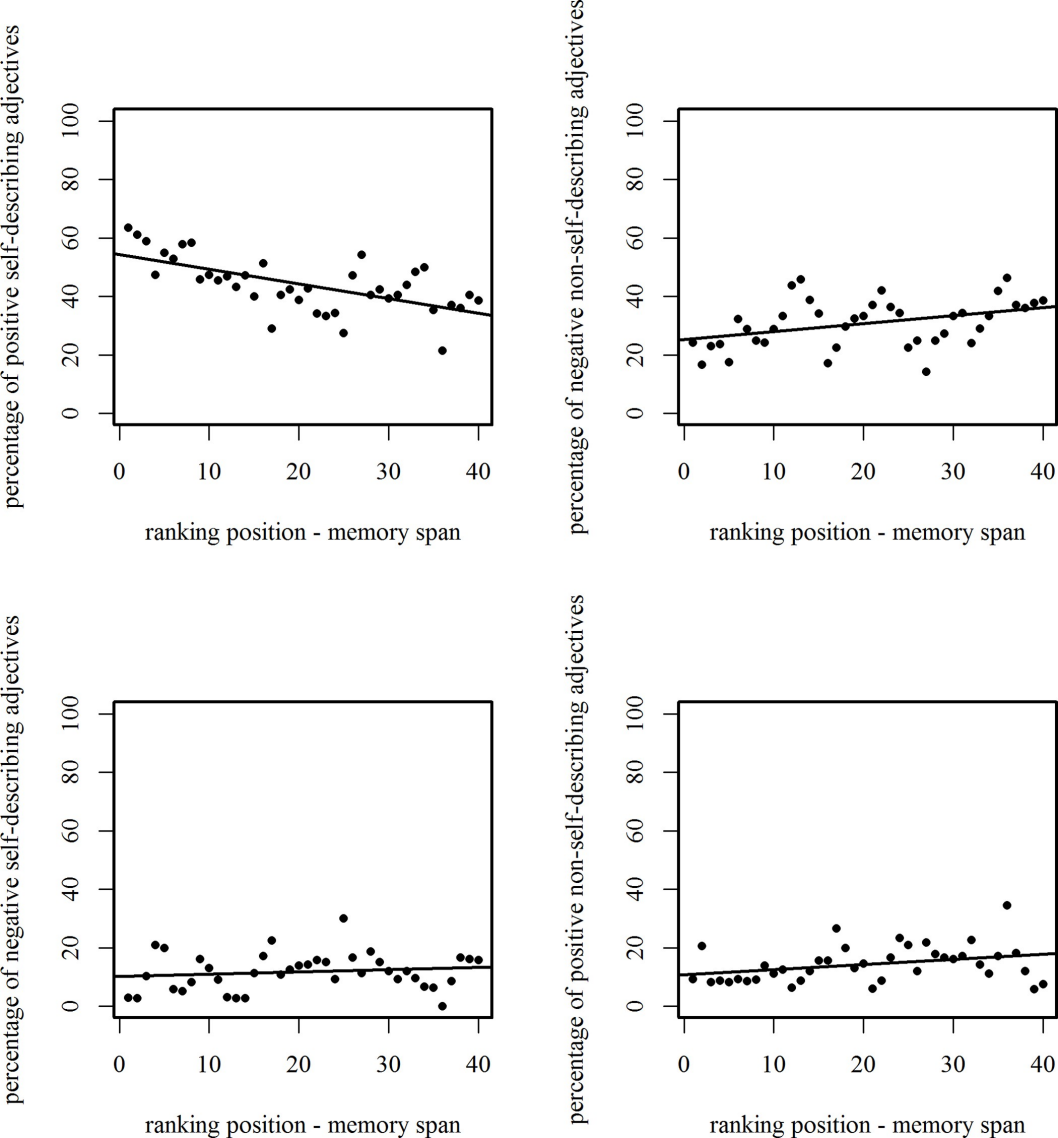
Associations between choices and memory ranks. Correlations between the percentages of adjectives chosen for each memory rank and the memory ranks (*N* = 57 for the percentage of positive self-describing, negative self-describing and negative non-self-describing adjectives; *N* = 53 for the percentage of positive non-self-describing adjectives).

The analysis of the association between memory ranks, self-appraisals, and valence (see Fig 4) revealed a significant correlation between the percentage of positive self-describing adjectives that were recalled (*M* = 44.1, *SD* = 9.4) and the memory ranks (1–40) (*r* = -.63, *p* < .001). This indicated that the recall of positive self-describing adjectives decreased across time. It also showed a significant correlation between the percentage of negative non-self-describing adjectives that were recalled (*M* = 30.8, *SD* = 8.2) and the memory ranks (*r* = .43, *p* = .005), indicating that the recall of negative non-self-describing adjectives increased across time. Furthermore, the percentage of positive non-self-describing adjectives (*M* = 14.4, *SD* = 6.2) was significantly correlated with the memory ranks (*r* = .32, *p* = .046), indicating that the recall of positive non-self-describing adjectives increased across time. However, the percentage of negative self-describing adjectives (*M* = 11.8, *SD* = 6.3) was not significantly correlated with the memory ranks (*r* = .16, *p* = .31).

### Differences between SELF-perspective and OTHER-perspective

From all of the positive and all of negative adjectives, 19 percent and 18.7 percent, respectively, were assigned differently in the two testing-conditions. The difference in assignments referred to one’s own self-appraisals versus reflected appraisals that another person was assumed to have.

#### Trait-judgment task

The ANOVAs for evaluating the impact of participants’ perspective on the appraisals revealed a significant effect for Diff_positive_ between the testing-conditions [*F*(1,56) = 5.74, *p* = .02,*η*^*2*^_*P*_ = .09, 90% CI(.01,.22), *η*^*2*^_*G*_ = .03] but not for Diff_negative_ [*F*(1,56) = 1.33, *p* = .25, *η*^*2*^_*P*_ = .02, 90% CI(0,.12), *η*^*2*^_*G*_ = .00]. This indicates that only the choices in the SELF and OTHER testing-condition for the positive adjectives were significantly different.

#### Trait-recall task

The repeated-measures ANOVA for the percentage of positive and negative adjectives that were recalled and previously assigned equally or differently in the two testing-conditions revealed a significant main effect for the valence of the adjectives [*F*(1,49) = 14.69, *p* < .001,*η*^*2*^_*P*_ = .23, 90% CI[.08,.38], *η*^*2*^_*G*_ = .04], but no significant main effect for the equality of the judgments [*F*(1,49) = 0.25, *p* = .62, *η*^*2*^_*P*_ = .01, 90% CI(0,.08), *η*^*2*^_*G*_ = .00] and no significant interaction [*F*(1,49) = 0.1, *p* = .76, *η*^*2*^_*P*_ = .00, 90% CI(0,.06), *η*^*2*^_*G*_ = .00]. This indicates that there were no differences between equally and differently assigned adjectives in self-appraisals and reflected appraisals in MNE. The means and standard deviations for the percentage of adjectives recalled which were assigned equally and differently in the two testing-conditions and post hoc pairwise comparisons of the group means are shown in Table 3.

**Table 3:** Summary of the recall performance for equally and differently assigned adjectives in the trait-judgment task.

Percentages of recalled adjectives assigned equally vs. differently (trait-recall task, *N* = 50)											
								Equality			
								equal		Different	
		Task						Valence			
		Judgment		Recall		Recall		POS^*a*^	NEG^*a*^	POS^*a*^	NEG^*a*^
Equality	Valence	*M* *(total)*	*SD (total)*	*M (total)*	*SD (total)*	*M* *(%)*	*SD (%)*	*p*^*1*^	*p*^*1*^	*p*^*1*^	*p*^*1*^
equal	POS^*a*^	19.6	2.5	6.9	2.3	35	11		.003	1	.39
	NEG^*a*^	20.1	2.5	5.6	2.5	27.5	11.6	.003		.03	1
different	POS^*a*^	5	2.2	1.8	1.4	37.2	26.9				.28
	NEG^*a*^	4.7	2.5	1.4	1.3	28.1	25.5			.28	

*Note*. Means and standard deviations for the total number and the percentage of recalled positive and negative adjectives which were assigned equally and differently in the two testing-condition in the trait-judgment task and the corresponding post hoc analyses.

^*1*^ Bonferroni-corrected *p* values

^*a*^ POS: positive, NEG: negative

### Association between self-esteem and choice

**Fig 5:**
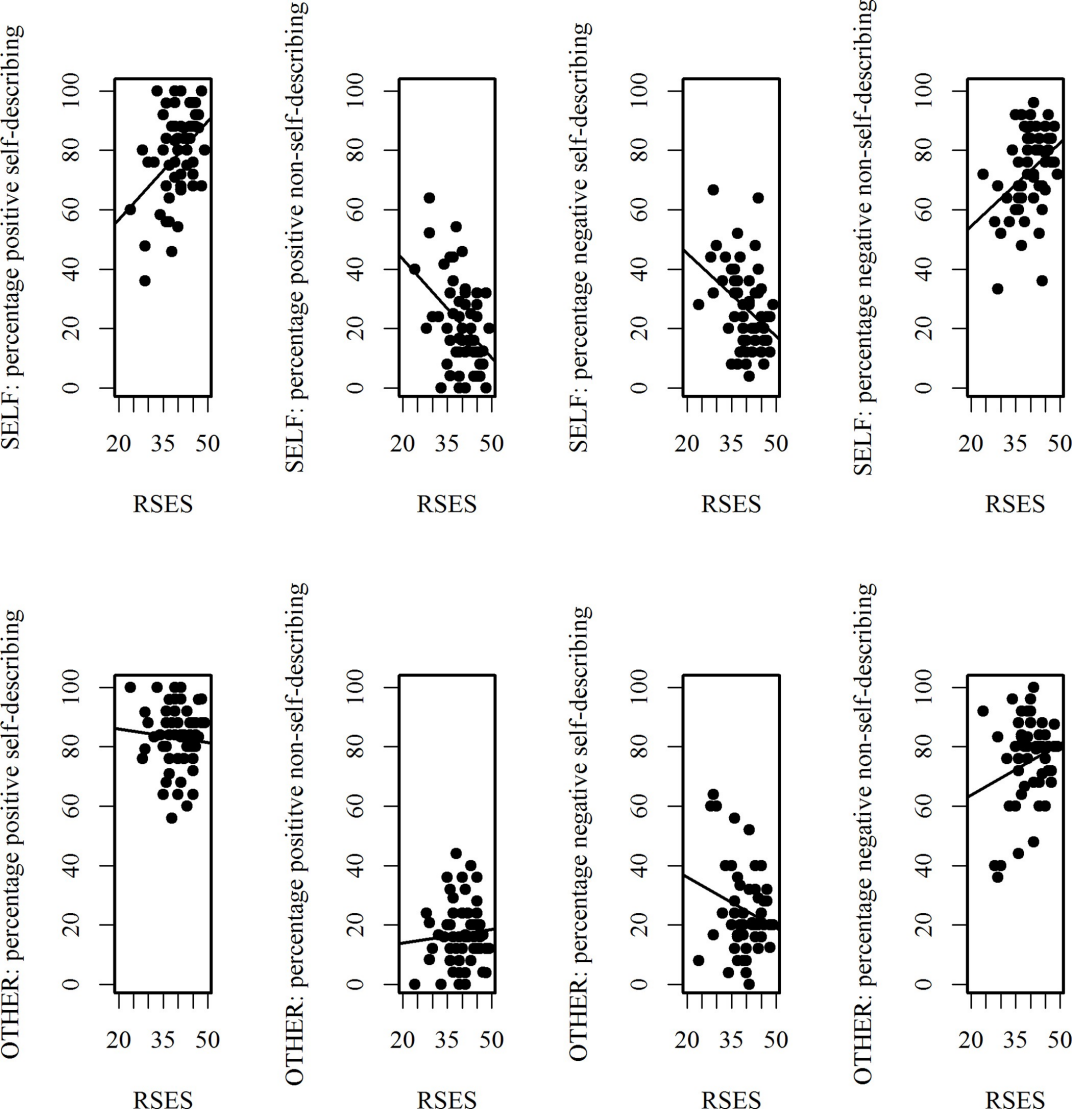
Associations between self-esteem and choices in the trait-judgment task. Correlations between self-esteem (sum scores on the Rosenberg Self-Esteem Scale (RSES); higher values on the x-axis indicate higher self-esteem, lower values on the x-axis indicate lower self-esteem) and the percentages of adjectives chosen as self-describing or non-self-describing in the SELF and OTHER conditions in the trait-judgment task.

Results of the regression analyses in which the sum scores from the RSES (*M* = 39.8, *SD* = 5.5) were the predictor variable and the percentages of self-describing and non-self-describing positive and negative adjectives in the SELF and OTHER testing-conditions were the dependent variables are displayed in Table 4 and Fig 5. Self-esteem was associated with the percentage of adjectives chosen in the SELF but not in the OTHER testing-condition. As self-esteem increased, more positive adjectives were selected as self-describing and more negative adjectives were selected as non-self-describing during the self-appraisals; by contrast, these judgments were relatively stable during the reflected appraisals.

**Table 4:** Regression analyses of the association between participants’ self-esteem and their choices in the trait-judgment task.

Percentages of chosen adjectives (trait-judgment task, *N* = 57)										
Allocation	Testing-condition	Valence	*R*^2^	*b*	*SE b*	*t*	*F*	*df*_1_	*df*_2_	*p*^*1*^
self-describing	SELF	POS^*a*^	.17	1.1	.32	3.4	11.57	1	55	.01
		NEG^*a*^	.14	-.93	.32	-2.96	8.73	1	55	.04
	OTHER	POS^*a*^	.01	-.15	.25	-.59	0.35	1	55	4.46
		NEG^*a*^	.05	-.57	.35	-1.64	2.69	1	55	.85
non-self-describing	SELF	POS^*a*^	.17	-1.1	.32	-3.4	11.57	1	55	.01
		NEG^*a*^	.14	.93	.32	2.96	8.73	1	55	.04
	OTHER	POS^*a*^	.01	.15	.25	.59	0.35	1	55	4.46
		NEG^*a*^	.05	.57	.35	1.64	2.69	1	55	.85

*Note*. Regression analyses with the sum scores from the Rosenberg Self-Esteem Scale as the predictor variable and the percentages of chosen adjectives as the dependent variables (*N* = 57).

^*1*^ Bonferroni-corrected *p*-values

^*a*^ POS: positive, NEG: negative

## Discussion

Because the concept of self is essential for psychological functioning and behavior in a goal-directed and socially appropriate manner, we examined self-referential processes to gain a better understanding of how one’s self-concept is formed, enhanced, and protected. We were specifically interested in exploring how an assumed opinion of a significant other differs from people’s own opinion about themselves. Prior research has confirmed that significant others do influence how people feel about themselves [10, 15]. Nevertheless, the overlap between self-appraisals and reflected appraisals of one’s own personality traits is not perfect [12]. In order to investigate the influence of two different perspectives (first person and third person) on appraisals of one’s self, two effects that have already been identified, SSBE and MNE, were examined in greater detail. Additionally, we also analyzed the association between reaction time and SSBE, which has been less frequently considered in previous studies, but which we introduced as SSRTE. Finally, we investigated how one’s self-esteem is related to self-appraisals versus reflected appraisals.

In the following sections, we first summarize our basic findings on the effects of SSBE, SSRTE, and MNE. Second, we discuss potential recall strategies that individuals might have used when forming the MNE. Third, we focus on our main findings concerning the differences between self-appraisals and reflected appraisals. Finally, we discuss what we learned about the association between self-esteem and self- and reflected appraisals.

Consistent with previous studies (e.g., [7, 19]), the results from the trait-judgment task revealed that participants assigned more positive than negative traits to themselves (SSBE), thus reflecting a typically mainly positive self-concept that healthy participants have. In line with this positive self-serving bias and the results of previous studies analyzing reaction time effects [21, 22], the participants took less time to make self-serving judgments, i.e., while ascribing positive and denying negative adjectives as self-describing compared to rejecting positive and ascribing negative adjectives (SSRTE). This suggests easier accessibility of information that accords with and supports one’s positive self-concept; i.e., adjectives that are congruent with a positive self-concept can be retrieved more easily. Moreover, the findings of the trait-recall task demonstrated a memory advantage for positive (not self-threatening) compared to negative (self-threatening) information assigned to one's own person (MNE), as Sedikides and Green [23] and Sedikides et al. [24] previously found. Participants recalled more adjectives that were not self-threatening (i.e., positive adjectives previously ascribed as self-describing and negative adjectives previously ascribed as non-self-describing) than self-threatening adjectives (positive adjectives previously ascribed as non-self-describing and negative adjectives previously ascribed as self-describing). Therefore, a stable, mainly positive self-concept in healthy people seems to be protected through an advantage in memory of adjectives that are not self-threatening. Altogether, these effects demonstrate the general tendency of healthy people to favor information that helps them form and protect a positive self-concept. With regard to the stability of SSBE found in many studies that tested healthy individuals, it should be noted that the term *self-serving bias* is actually inappropriate, inasmuch as the self-serving aspect is more descriptive of something that is general, systematic, or naturally occurring than a tendency to deviate from the norm. Regarding the SSBE as representing the norm, we suggest it would be sufficient to refer to this effect solely as a *Self-Serving Effect* (SSE).

As Newman et al. [25] pointed out, the MNE can be affected by, for example, the length of the recall period and the recall procedure used. Thus, we analyzed specific recall strategies that the participants might have used. The first analysis indicated that participants most frequently recalled positive adjectives that had been allocated as self-describing more than once, and these were followed by negative adjectives that had been previously allocated as non-self-describing. Again, this indicates a greater accessibility of information that is not self-threatening. Another analysis indicated that the self-relevance of information is more important for the accessibility of information than is stimulus valence. From the correlational analyses of the associations among the availability in memory and the self-descriptiveness and valence of the adjectives that were recalled, it is evident that positive self-describing adjectives were best remembered at the beginning of the recall task and negative non-self-describing adjectives were best remembered at the end of the recall task. Newman et al. [25] suggested that information that is highly accessible is also recalled best. After the highly accessible information has been recalled, the memory search mode becomes more effortful. From our own results, it appears that positive self-describing adjectives were the most accessible. However, after a period of time had elapsed, the positive self-describing adjectives were less well remembered, but the negative non-self-describing adjectives were remembered better. Therefore, it seems plausible that during an effortful memory search participants still favored not self-threatening adjectives, even if they were unable to recall as much additional positive self-describing information as they had previously. One could also speculate that positive self-describing adjectives were initially remembered better, because people tend to strive for a positive self-concept; therefore, they tend to recall positively valenced items better. In summary, participants’ recall strategy seemed to be to first recall information that supported their positive self-concept; thereafter, they recalled information that simply did not threaten their positive self-concept. Furthermore, their recall strategy might have been an overall preference for not self-threatening information, because both positive self-describing and negative non-self-describing adjectives were always remembered better than positive non-self-describing or negative self-describing adjectives. Taken as a whole, the results support the assumption that self-relevance rather than pure stimulus valence enhances recall availability and thereby reflects a strategy that preserves a positive self-concept during recall.

The main aim of this study was to investigate the differences between self-appraisals and reflected appraisals. Carlson et al. [6] had already shown that people are able to adopt another person’s perspective. However, the present study aimed not simply to confirm the accuracy of perspective taking, which would imply that we could verify the accuracy of the reflected appraisals of another person’s judgments as true or false. Instead, our study examined the extent to which self-appraisals differ from other kinds of appraisals. Consistent with the findings reported in the prior literature (e.g. 12, 13, 50]), the frequency with which participants chose the presented adjectives to be self-describing or not in the two appraisal perspectives was not equal. Instead, 19 percent of all of the positive adjectives and 18.7 percent of all of the negative adjectives were chosen differently in the two testing-conditions. The analysis of the differences with which self-describing and non-self-describing adjectives were chosen in each testing-condition showed a significant difference between the SELF and the OTHER testing-conditions in the choice of positive adjectives. This means that when adopting the perspective of a significant other (OTHER-condition), significantly more positive adjectives were chosen as self-describing. There was also a trend for negative adjectives to be chosen at a different frequency when they were self-describing in the SELF-condition compared with the OTHER-condition. Importantly, the patterns of interest (in SSBE, SSRTE and MNE) were found in both of the testing-conditions. However, SSBE was somewhat more pronounced during reflected appraisals. It seems that people assume that others see them in an even more positive light than they see themselves and that they assume others to focus more on their positive as compared to their negative traits. One could speculate that this finding emerges through people’s general tendency and motives to self-enhance and protect. Moreover, participants might have reflected how they themselves would rate a significant other person thereby assuming a similar strategy, namely adapting the appraisal to a normative personality profile which, in general, is rather positive [51]. Furthermore, it is known from previous studies that significant others actually do report a positive biased appraisal of the participants whom proposed them as significant other [52]. Therefore, it could be, that participants might have previously provoked and actually received self-enhancing feedback from the selected significant other [53], an impression, which may have strengthened the belief that the other person has an even more positive impression of them than they do themselves in general. Another explanation for the study finding, that participants assumed that close others assess them even more positively than they themselves do could be the cognitive load associated with the OTHER-condition. Study results by Hixon and Swann [54] indicate that self-enhancing appraisals are increasingly made under cognitive load. In the OTHER-condition of the study at hand, the participants were not only under time pressure, but also had to perform a change of perspective, whereby the cognitive load in this condition was probably higher than in the SELF-condition. Indeed, participants needed significantly more time to form reflected appraisals compared to forming self-appraisals, but, importantly, this difference was equal in all of the testing-conditions (self-describing positive, non-self-describing positive, self-describing negative and non-self-describing negative) and hence is best explained by the additional cognitive effort that was involved in adopting another person's perspective. With regard to recall, both the adjectives that were equally assigned in the judgment task and those that were differently assigned were remembered equally well; i.e., neither equally nor unequally assigned adjectives had a subsequent advantage in participants’ memory. In conclusion, we suggest that the differences between self-appraisals and reflected appraisals do not have an impact on one’s memorized self-concept.

Finally, we assessed the extent to which self-esteem is an important factor in the maintenance of a stable, primarily positive self-concept and how this was related to participants’ choices during the self-appraisals and reflected appraisals. Consistent with the conclusion of Asencio [36] that a person’s self-esteem seems to moderate the influence of reflected appraisals on one’s self-appraisal, the present study found that self-esteem was significantly associated with choices in the self-appraisals (between 14 and 17 percent of the variance in self-appraisals could be explained by participants’ own self-esteem) but not in the reflected appraisals. As one’s self-esteem increases, SSBE became stronger from the perspective of the first person, whereas this effect was relatively consistent when participants adopted another person’s perspective. In addition, participants with lower self-esteem were more likely to judge themselves more negatively in the self-appraisals than in the reflected appraisals. Because SSBE occurs in both kinds of perspectives and considering that self-esteem is associated with SSBE in self-appraisals, we assume that participants with higher self-esteem have a more stable self-concept, i.e., as reflected in their choice of equally more positive adjectives as self-describing and equally more negative adjectives as non-self-describing in both of the testing-conditions. In conclusion, self-esteem seems to have a positive impact on the stability of one’s self-concept in that mostly congruent information is taken into account in self- and reflected appraisals.

### Limitations and future directions

We would like to make a few remarks concerning possible study restrictions and how our findings might inspire further research questions. First, we presented the participants with the same stimuli twice in order to be able to compare the choices made in self-appraisals and reflected appraisals. This, however, might have enhanced participants’ recall. We cannot, therefore, rule out that the possibility that the MNE was more pronounced in our study than in other studies that did not include a change in perspective or did not present the same stimuli twice. Moreover, to control for primacy and recency effects in participants’ memory, we excluded the first and the last 10 trait-judgments from the final analyses [55, 56]. This might have decreased participants’ recall in comparison to studies without such restrictions. More generally, the fact that we introduced a recall task at all led to a dichotomous scale on which the participants rated the personality traits. This forced them to make rather extreme choices, although certain personality traits might be rated more accurately on a broader rating scale.

Second, the results of this study suggest that people, especially people with lower self-esteem, assume that other people who are close to them view them even more positively than they view themselves. This finding, as well as the finding that self-assessments and actual external assessments and reflected appraisals do not perfectly coincide [12], raises the question as to which other factors could cause this to happen. As this study demonstrates, a person’s self-esteem seems to be an influencing factor although only 14 to 17 percent of the variance in self-appraisals could be explained by differences in self-esteem. Other factors, such as how close the participant is to the third person, should be examined further. Previous studies that performed a trait-judgment task without a change of perspective have shown that both SSBE and MNE are affected by the other person who is being judged on in the comparison condition. In particular, how close the participant feels to the other person has an impact on the effects that are being studied, as other people with whom the participant is highly intimate lead to significantly smaller effect than others with whom the participant is less intimate [27]. This phenomenon might also account for the results of studies that have included a change in perspective, i.e., when people rate themselves from the perspective of a third person. A study by Li et al. [29] demonstrated that in reflected appraisals (i.e., involving a change in perspective), the perceived closeness between a person himself or herself and the other person whose perspective is being taken might modulate the neural basis for SSBE. This neural modulation should also be reflected in corresponding behavioral changes. Further, previous studies have shown that the information that other people have about a person varies according to how close they are to that person [57]. Old friends know different things about each other compared to what strangers know, and this leads to different assumptions about what significant others are assumed to be thinking [6, 57]. In the present study, a person with whom the participant was highly familiar was deliberately chosen for the OTHER-condition, as we aimed to start by studying differences in self-appraisals and reflected appraisals among people who knew each other very well. This might explain the large overlap between the different kinds of appraisals in the present study. We suggest, therefore, that future studies vary the closeness between the participant and the other person whose perspective is to be adopted. Doing so would allow the researcher to investigate the influence of closeness on perspective taking and also the importance of the other person’s knowledge for the participant's own self-concept. It would be interesting to take an actual significant other person’s perspective into account, for then the overlap between the appraisals and the meta-accuracy could be determined.

Third, only healthy female participants were included in the present study. We deliberately chose a homogeneous sample in order to minimize random error and to rule out confounding effects. For this reason, however, the results might be biased, for it would seem likely that men and women differ in their ability to adopt another person’s perspective. O'Brien et al. [37] showed, in fact, that men and women differ in their self-reported perspective taking. Also, men tend to have a stronger self-esteem than women [38], and this would be expected to have an impact on the effects that we identified. Future studies should, therefore, take potential gender differences into account. Moreover, it would also be important to determine how the effects identified in the current study would manifest themselves in a pathogenic sample of participants with very low or very high self-esteem and what difficulties such individuals might have in perspective taking. For example, patients with a depressive disorder have very low self-esteem [58] and would be assumed to have difficulties in accurately adopting another person’s perspective [59, 60]. Because of these potential difficulties, it seems plausible to assume that there would be little difference between their self-appraisals and reflected appraisals. Still, as just indicated, patients with a depressive disorder most often report a low self-esteem, which in the present study was associated with differential assessments from the perspective of a first person and a third person. Another example would be patients with a narcissistic personality disorder who are characterized as having an extremely positive self-concept [61]. Patients with a narcissistic personality disorder often experience difficulties in social interactions, assumingly because of their lack of empathy and the difficulties they have in assuming another person’s perspective [62]. Considering that some patients have distorted self-images (i.e. either overly positive or overly negative) and that treatment might be aimed at altering these people’s self-concept, future studies should investigate self-referential processes from differing perspectives and whether a person’s self-esteem has an impact on these processes, particularly in patients with psychological disorders. Doing so could improve our understanding of how patients’ self-concept is formed and how it interacts with multiple other factors, such as the ability to assume different perspectives and self-esteem.
